# Microbial and Genomic Information Synergistically Contribute to Predicting Swine Performance Across Production Systems

**DOI:** 10.1111/jbg.70014

**Published:** 2025-09-24

**Authors:** Christian Maltecca, Enrico Mancin, Jicai Jiang, Maria Chiara Fabbri, Riccardo Bozzi, Clint Schwab, Francesco Tiezzi

**Affiliations:** ^1^ Department of Animal Science North Carolina State University Raleigh North Carolina USA; ^2^ Dipartimento di Scienze e Tecnologie Agrarie, Alimentari, Ambientali e Forestali Università di Firenze Firenze Italy; ^3^ Dipartimento Agronomia Animali Alimenti Risorse Naturali e Ambiente (DAFNAE) Università degli Studi di Padova Legnaro Italy; ^4^ AcuFast LLC Navasota Texas USA

## Abstract

Microbiota composition represents a promising tool in precision farming, simultaneously serving as a benchmark of environmental challenge, a predictor of animal physiological status, and a direct target for host selection. In this paper, we compared the ability of microbiota composition and genomic information to predict swine performance in two production settings, namely a purebred nucleus (NU) and a terminal cross commercial population (TE). Microbiota consistently predicted all traits in both scenarios (NU‐TE: training on NU to predict TE; TE‐NU: training on TE to predict NU) and at two time points: mid‐test and off‐test. The highest correlation (i.e., prediction accuracy) was achieved for back fat, with values of 0.08 and 0.04, and 0.30 and 0.23 for mid and off‐tests, predicting from nucleus to terminal, and vice versa. Similarly, daily gains correlations were 0.05 and 0.04, and 0.18 and 0.15 for the same time points and scenario combinations. Including genomic information yielded correlations ranging from low for loin area to moderate for back fat (0.19 nucleus to terminal, 0.16 for the opposite). Microbiota had higher prediction accuracies than genomic for back fat both from nucleus to terminal and vice versa (+0.11, +0.07) and daily gain (+0.08, +0.02) at off‐test. Lower accuracies were obtained for the IMF. Including genomic and microbial information produced higher accuracies than microbiota or genomic alone for back fat (0.37 and 0.29 for nucleus to terminal and opposite) and daily gain (0.19 and 0.21 for nucleus to terminal and opposite). Results for other traits differed for different scenarios. Results show that microbiota composition effectively predicted most growth and carcass traits, particularly growth and fat deposition, across production systems, prediction scenarios (NU‐TE and TE‐NU), and time points (mid‐test and off‐test). These findings highlight the potential of microbiota profiles to predict phenotypes across production systems and support their use as a tool for selecting animals in environments they have not been exposed to.

## 1 Introduction

Despite the growing attention directed towards the gut microbiota and its potential applications in agriculture, there remain significant gaps in our understanding of the interactions between hosts and microbes and how their interactions influence the phenotype expression. The investigation of microbial communities and their fluctuations over time, across different environmental conditions, and under various management practices is a thriving area of research in livestock studies (Gardiner et al. 2020; Le Sciellour et al. 2019). Microbial sequencing data are inherently rich in information, serving simultaneously as an indicator of ecological status (Ramayo‐Caldas et al. 2020), a predictor of the physiological state of animals (Wang et al. 2020, 2019), and a potential target for direct host selection (Camarinha‐Silva et al. 2017; Pérez‐Enciso et al. 2021; Bergamaschi, Maltecca, Schillebeeckx, et al. 2020). Consequently, numerous recent studies have been conducted to investigate the role of microbial composition in influencing various factors. Recent scientific studies have established connections between the microbiota and host processes such as drug metabolism (Swanson 2015; Kim 2015), physiological development throughout growth (Le Sciellour et al. 2019), growth efficiency (Martinez‐Alvaro et al. 2021; Weishaar et al. 2020; Quan et al. 2018), reproductive performance (Sanglard et al. 2020), responses to dietary changes (Sciellour et al. 2018), and even the possibility of enhancing genetic variability through selection (Tiezzi et al. 2021).

The versatility of abundance measures holds significant promise for collecting and utilising microbial information in livestock applications, but it also presents distinct challenges. Among these challenges, one of the most crucial aspects pertains to the applicability of results. Most investigations conducted thus far have been on a smaller scale, often within research environments, and adopting a reductionist approach that evaluates a limited number of treatments at any given time. While this approach is advantageous for testing specific interventions or manipulations (e.g., dietary supplements), it becomes limiting when utilising microbial information to predict future outcomes (related to individual health or production) within larger breeding populations. This limitation is particularly pertinent in the context of pigs, given the structural divide in the industry, where distinct management and genetic compositions exist between nucleus selection and terminal market populations.

At least in principle, the microbiota has the potential to help recover information both within and between production systems by recapitulating non‐measured descriptors of environmental variation. Moreover, if a common genetic factor influences the formation and stability of host‐associated microbial communities, this variability could be harnessed in selection programs to sustain selection pressure (Maltecca et al. 2020; Verschuren et al. 2020).

Our current knowledge regarding the variability of the microbiota across distinct swine production systems remains limited. In a previous study conducted by our research group, we undertook an ecological assessment to elucidate the microbial composition disparities among different production schemes (Maltecca et al. 2021).

In our study, we are interested in how well microbial abundance counts and genetic information can predict the performance of pigs in different farming systems. We are particularly interested in using data from terminal or nucleus populations to predict how pigs will perform in the other type of system.

## 2 Materials and Methods

### 2.1 Experimental Design and Data Collection

Phenotypic records presented in this study were obtained from a commercial and a nucleus farm operated by The Maschhoffs LLC (now Acuity, Carlyle, IL, USA). All methods and procedures followed the Animal Care and Use policies of North Carolina State University and the National Pork Board. The experimental protocol for faecal sample collection received approval number 15027 from the Institutional Animal Care and Use Committee. All pigs were harvested in commercial facilities under the supervision of the USDA Food Safety and Inspection Service.

The data spanned two connected systems: a Duroc nucleus purebred population (NU) and a terminal commercial crossbred population (TE), both sired by 28 Duroc founding boars. Identification, sex, cross‐fostering status, litter, sow identification, and parity were collected for all individuals in the experiment. Sex was equally distributed among pig populations.

The NU population consisted of 769 Duroc individuals (387 males and 387 females). Individuals were raised in a fixed‐time system. Individuals entered the performance test at 88.50 ± 9.92 days of age and were taken off test at 178.4 ± 7.96 days of age (avg. 129.49 ± 17.72 kg of weight). The TE population consisted of 1141 individuals (565 females and 576 castrated males) generated by crossing the Duroc sires with two commercial sow lines (Yorkshire × Landrace and Landrace × Yorkshire). Crossbred commercial individuals were raised in a fixed‐weight testing system and harvested at an average weight of 98.8 ± 10.19 and 97.90 ± 7.63 kg for the males and females, respectively. In both systems, a contemporary group was defined as the group of animals that entered a given facility simultaneously. Individuals were allocated in single‐sire, single‐sex groups of 20 heads and housed in the same pen. Feed and water were provided ad libitum to pigs.

Details of diets, their nutritional values, and standard vaccination and medication routines can be found in Maltecca et al. (2021). Rectal swabs were collected from all pigs at three time points: weaning (S1; as described above for NU, average 90.63 ± 1.57 days for TE), mid‐test (S2; average 118.20 ± 1.18 days for TE and 116.30 ± 2.3 for NU), and off‐test (S3; as described above for NU and 176.45 ± 1.82 days for TE).

### 2.2 Microbial Sequences Bioinformatics and Processing

#### 2.2.1 16S rRNA Gene Sequencing

DNA extraction, purification, Illumina library preparation, and sequencing were done as described by Lu et al. (2018). Briefly, total DNA (gDNA) was extracted from each rectal swab by mechanical disruption in phenol:chloroform:isoamyl alcohol solution. Bead‐beating was performed on the Mini‐BeadBeater‐96 (MBB‐96; BioSpec, OK, USA) for 4 min at room temperature, and samples were centrifuged at 3220 *g*. The DNA was then purified using a QIAquick 96 PCR purification kit (Qiagen, MD, USA), with minor modifications to the manufacturer's protocol. Changes included the addition of sodium acetate (3 M, pH 5.5) to Buffer PM to a final concentration of 185 mM, combining crude DNA with four volumes of Buffer PM, and elution of DNA in 100 μL of Buffer EB. All sequencing was performed at the DNA Sequencing Innovation Laboratory at the Center for Genome Sciences and Systems Biology at Washington University in St. Louis. Phased, bi‐directional amplification of the V4 region (bases 515–806) of the 16S rRNA gene was employed to generate indexed libraries for Illumina sequencing, as described in Faith et al. (2013). Sequencing was performed on an Illumina MiSeq instrument (Illumina Inc., San Diego, USA), generating 250 bp paired‐end reads.

#### 2.2.2 Taxonomic Classification

16S rRNA gene sequencing and data quality control were conducted as described by Lu et al. (2018). Briefly, the pairs of 16S rRNA gene sequences obtained from Illumina sequencing were combined into single sequences using FLASH v1.2.11 (Magofç and Salzberg 2011). The sequences with a mean quality score below Q35 were filtered out using PRINSEQ v0.20.4 (Schmieder and Edwards 2011). Forward‐oriented sequences were searched for primer sequences, allowing up to 1 bp of mismatch, and primer sequences were trimmed. Sequences were subsequently demultiplexed using QIIME v1.9 (Caporaso et al. 2010). Although we acknowledge that amplicon sequence variants (ASVs) offer finer resolution of biological variation and may better capture functional differences within microbial communities (Callahan et al. 2017), our focus was on prediction accuracy rather than biological interpretation. While underlying microbial functions may still influence predictive performance, we chose to retain the current approach to ensure comparability with results presented by the same group in a previous study (Maltecca et al. 2021).

QIIME was used to cluster the nucleotide sequences into operational taxonomic units (OTUs) using open‐reference OTU picking as described by Lu et al. (2018). A modified version of Greengenes (DeSantis et al. 2006) was used as the reference database. Input sequences that had 10% of the reads with no hit to the reference database were then clustered de novo with UCLUST (Prasad et al. 2015) to generate new reference OTUs to which the remaining 90% of reads were assigned. The most abundant sequence in each cluster was used as the representative sequence for the OTU. Sparse OTUs were then filtered out by requiring a minimum total observation count of 1200 for an OTU to be retained, and the OTU table was rarefied to 10,000 counts per sample. Average good's coverage estimates for samples at weaning, Week 15, and off‐test were 0.99 ± 0.002, 0.98 ± 0.002, and 0.98 ± 0.002, respectively. Finally, the Ribosomal Database Project (RDP) classifier (v2.4) was retrained in the manner described in Ridaura et al. (2013) with a 0.8 cutoff used to assign taxonomy to the representative sequences.

### 2.3 Genomic Information

Genomic information on the 28 funding Duroc sires, the 1141 TE pigs, and the 769 NU pigs was obtained using the porcine single nucleotide polymorphism (SNP) 60 v2 BeadChip (Illumina Inc.). Standard quality control procedures were performed on the combined genomic data for TE and NU individuals, which removed non‐autosomal SNPs and SNPs with a call rate lower than 0.90 and/or minor allele frequency lower than 0.05. In total, 42,529 SNPs remained after quality control.

### 2.4 Phenotypic Information

Growth and carcass composition were collected in the two populations according to the standards of the respective production systems. This meant that phenotypes for the NU individuals were obtained at off‐test while at market weight for TE individuals.

#### 2.4.1 Terminal Population

Growth and carcass traits were measured on live animals at market weight. They included loin depth (MLD), back fat (MBF), intramuscular fat (MIMF), loin eye area (MLEA), and average daily gain (MADG). MBF, MLEA, and MLD were measured at slaughter with a Fat‐O‐Meater probe (SFK Technology A/S, Herlev, Denmark) at the approximate location of the 10th rib. The MADG was calculated by dividing the difference between the hot carcass weight and birth weight by the pig's age at slaughter. MIMF was determined as described by Wilson et al. (2016).

#### 2.4.2 Nucleus Population

Traits recorded in NU included intramuscular fat (OTIMF), backfat (OTBF), loin depth (OTLD), loin eye area (OTLEA), and average daily gain (OTADG). OTIMF, OTBF, OTLD, and OTLEA measures were collected using an ultrasound probe (Biotronics Inc., Ames, IA, USA). The OTADG was calculated by dividing the difference between the pig's off‐test weight and birth weight by the pig's age at the off test. This trait was labelled OADG to reflect that the age at off‐test differed from the age used in calculating MADG, and to indicate that it was measured in a purebred population.

A summary of the traits included in the analysis is provided in Table 1. Detailed phenotypic data collection protocols can be found in Khanal et al. (2020) and Bergamaschi, Maltecca, Fix, et al. (2020). Genetic correlations among the traits can be found in Table S1 and Lozada‐Soto et al. (2022).

**TABLE 1:** Descriptive Statistic of traits used in the analysis.

Trait	Unit	Acronym	Pop	Mean	Median	SD	min	max
Average daily gain	kg	MADG	TE	0.52	0.52	0.06	0.3	0.89
Average daily gain	kg	OADG	NU	0.87	0.87	0.12	0.38	1.36
Market backfat	cm	MBF	TE	2.35	2.29	0.64	0.84	5.46
Offtest backfat	cm	OBF	NU	1.59	1.57	0.35	0.51	3.22
Market intramuscular fat	%	MIMF	TE	2.71	2.61	0.93	0.44	7.23
Offtest intramuscular FAT	%	OIMF	NU	2.38	2.4	0.83	0.1	7.6
Market loin depth	cm	MLD	TE	6.03	6.02	0.51	3.91	8.05
Offtest loin depth	cm	OLD	NU	6.03	6.02	0.57	2.59	8.05
Market loin area	cm^2^	MLEA	TE	50.38	50.39	5.84	24.32	76.13
Offtest loin area	cm^2^	OLEA	NU	51.71	51.81	6.12	15.87	73.42

In the previous section, we have defined traits within systems. We did this to draw attention to the lower‐than‐unit correlations across the systems of the phenotype measures taken. However, we will present traits with identical labeling hereafter to facilitate the interpretation and comparison in the results and discussion sections.

### 2.5 Cross‐Validation

#### 2.5.1 Prediction Scenarios

In this study, we explored two distinct prediction scenarios. These two prediction scenarios were designed to evaluate the ability of microbiota and genomic data to predict phenotypes across distinct production systems, with sires as the only linking element reflected. Assessing predictive performance in both directions (NU‐TE and TE‐NU) allows us to investigate potential asymmetries in information transfer due to differences in population structure, management, and environmental conditions.

**FIGURE 1:**
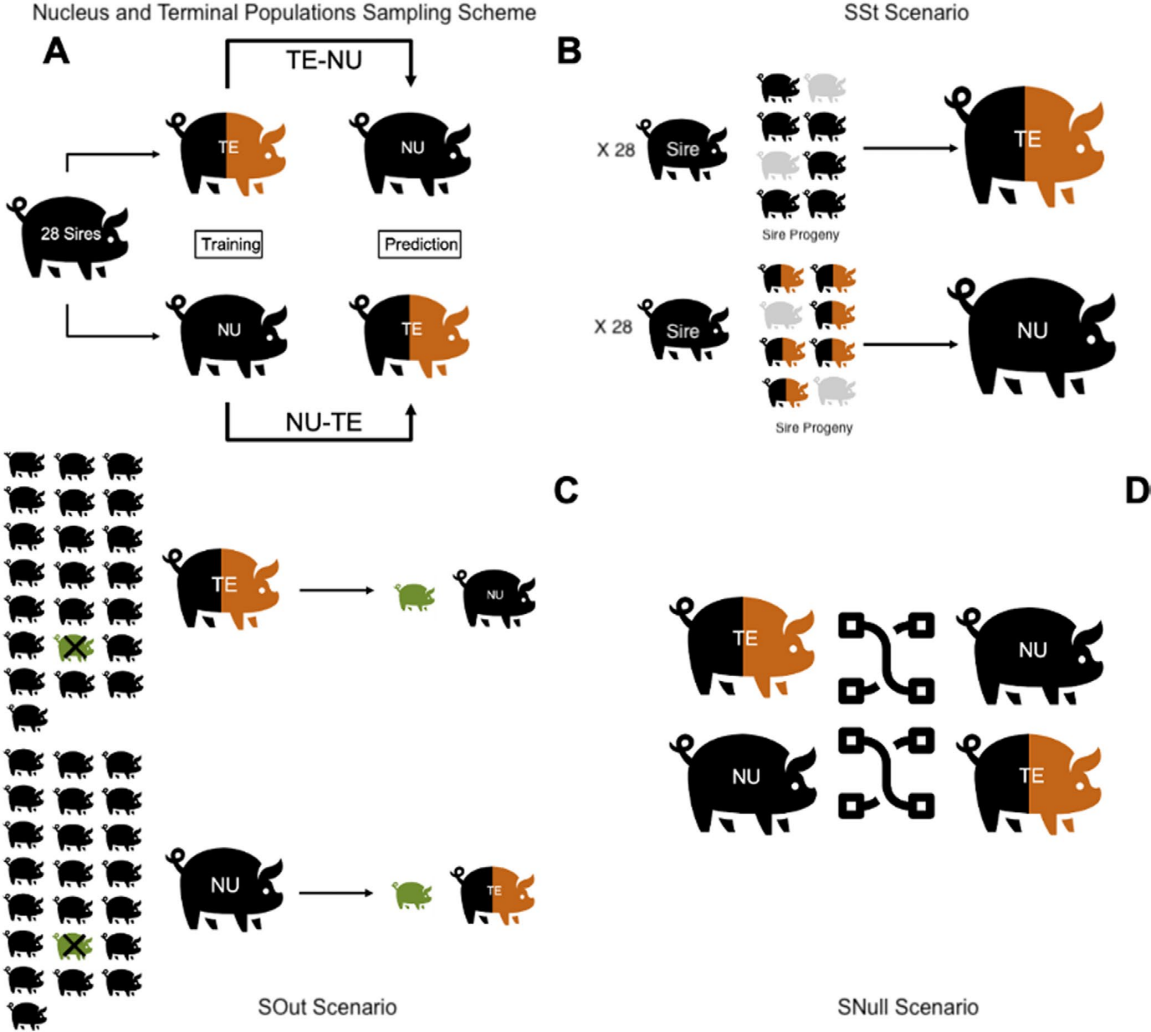
Cross‐validation scheme. (Panel A) General sampling scheme. Twenty‐eight funding generated individuals in the nucleus (NU) and terminal (TE) populations. In NU‐TE individuals in NU were used to predict individuals in TE; in NU‐TE individuals in TE were used to predict pigs in NU. (Panel B) SSt (Stratified Sire scenario). A fold was generated by excluding approximately 10 individuals from each sire family, preserving the family structure (with siblings included in training) while equally reducing the number of individuals per family. Process repeated 4 times. Models trained in each fold were used to predict the opposite population (NU or TE, respectively). (Panel C) SOut (sire out scenario). A fold was generated by excluding progeny of a sire in the training population. Accuracy of predictions was measured on progeny of the excluded sire in the validation population. This was repeated 28 times (the number of sires). (Panel D) SNull (null scenario). We repeated SSt and SOut but shuffling phenotypes within training populations (500 rounds). Note that TE pigs are shown in two colours to indicate breed composition, as they result from crosses between NU sires and dams of a different breed, highlighting the mixed genetic background within the TE population. [Colour figure can be viewed at wileyonlinelibrary.com]

Specifically, the first scenario involved training our predictive models on the NU population to make predictions for the TE population, a setup denoted as NU‐TE. In contrast, the second scenario focused on the TE population as the training dataset, with predictions for the NU population, denominated TE‐NU. It is important to note that sires were the only linking element between the two data sets. The average number of progenies per family for the 28 founding sires was 40.75 and 27.50 for TE and NU, respectively. A representation of these prediction scenarios is depicted in Figure 1A.

#### 2.5.2 Sampling Strategies

Within the prediction scenarios just described, we implemented three sampling strategies. In the first strategy, we employed a sire‐stratified cross‐validation approach, SSt hereafter. Within each training population (NU or TE, respectively), a fold was created, excluding ~10 individuals per sire family, such that the family structure (presence of sibs in training) was preserved, but the number of individuals per family was equally reduced. Consequently, we developed predictive models for each combination of omic data, measurement type, and time point; all combinations of analysis were reported on Table S2. The solutions derived from these models were then applied to predict the corresponding phenotypes within the prediction set, either NU or TE. It is important to note that, in this context, the prediction was obtained for the entire prediction set, which remained consistent throughout the procedure, unlike the conventional cross‐validation framework where the excluded fold is used in prediction. This process was iterated four times for each unique combination of factors. While we retained the family structure within the training data, the sole familial link connecting the training and prediction sets was provided by the common sire so that no full or half‐sibs were in common between training and prediction. A representation of this sampling strategy is reported in Figure 1B.

In our study, the TE and NU populations were spatially separated. However, within each pen, individuals in both populations belonged to single‐sire families, which could potentially cause confounding. To address this issue, we implemented a second sampling strategy called SOut. This strategy involved a cross‐validation approach where one sire family was left out during training, and the remaining families were used to predict the progeny of the excluded sire family in the validation. Specifically, within each training population (NU or TE), data from the progeny of one founding sire were excluded. Models were then trained as described previously, and predictions were made only for the progeny of the excluded sire in the validation population.

The cross‐validation was performed 28 times as the number of sires. A depiction of this sampling approach is illustrated in Figure 1C.

To address the potential influence of unaccounted spurious associations on predictions, we introduced a final sampling strategy called SNull. This procedure involved shuffling the phenotypes within each training population, fitting models for each factor combination, and using the solutions to predict the unshuffled validation population (Figure 1D). Permutations were repeated 500 times for each scenario (NU‐TE and TE‐NU) for the raw phenotypes while keeping all other factors constant, and a null distribution of expected prediction values was created. The family structure of SSt and SOut was preserved in this step so that the SNull was repeated twice. Since there was no substantial difference between the two replicates, results were pooled, and results will be presented together.

### 2.6 Models

Predictions were obtained fitting microbial composition and genomic information using a Bayesian Ridge Regression model fitted through the BGLR R package (Pérez and de los Campos 2014). The models for NU and TE were similar.

For all three microbial sampling time points (S1, S2, S3), we fitted three separate models: a model including only microbial information (M) of the following form

$$y=\text{Fixed}+\text{Micr}o_{s}+\text{Rand}+e$$

a model including only genomic information (G) of form

y=Fixed+Geno+Rand+e

and a model including both sources of information (M + G) of the form

$$y=\text{Fixed}+\text{Micr}o_{s}+\text{Geno}+\text{Rand}+e$$

where *y* is one of the phenotypes in the analysis, the subscript *s* corresponds to the time at sampling for the Microbiota (S1, S2, and S3), and Fixed and random Rand are the fixed and random effects (other than microbiota and genomic information) outlined below:

In TE, models fixed effects included sire (28 levels fitted solely for models including only microbial information), contemporary group (12 levels), maternal genetic line (2 levels), and the random effects of pen (which here was the physical group of same‐sex paternal half‐sibs), and the residuals.

In the NU population, fixed effects included sire (28 levels fitted solely for models including only microbial information), contemporary group (66 levels), sex (2 levels), and the random effects of the biological litter where the individual was born, plus the residual error.

Microbial composition was included as $\sum_{1}^{3001}m_{i}X_{i}$ where $m_{i}$ is the OTU effect (3001 OTUs), $X_{i}$ is a column vector of a matrix of centered‐log‐ratio transformed OTU counts obtained with the *crl()* function of the composition R package (van den Boogaart et al. 2021).

Genomic information was included as $\sum_{1}^{42529}g_{i}Z_{i}$ where $z_{i}$ is the SNP effect, and $Z_{i}$ is a column vector of a matrix SNP allele counts (0,1,2).

Distributional assumptions were: $\text{litter}\sim N\left(0,I\sigma_{\text{litter}}^{2}\right)$, $\mathrm{pen}\sim N\left(0,I\sigma_{\mathrm{pen}}^{2}\right)$ $m\sim N\left(0,I\sigma_{m}^{2}\right)$ $g\sim N\left(0,I\sigma_{g}^{2}\right)$ and $e\sim N\left(0,I\sigma_{e}^{2}\right)$. All prior variances were assigned a scaled‐inverse Chi‐square prior density. BGLR provides a convenient way to choose priors shape through the R2 flag. R2 can roughly be interpreted as the expected variance proportion explained by the effect included in the model. For all random effects, default degrees of freedom of 5 were employed, and an R2 of 0.1 was employed for all random effects except for genomic information 0.3 and residuals 0.5. Prior scale parameter was then obtained as, $\mathrm{Sp}=\mathrm{Var}\left(y\right),\left(1-R2\right),\left(\mathrm{dfp}+2\right)$ with Sp and dfp the scale and degrees of freedom, respectively.

A single chain of 100,000 iterations was run for all models, with 30,000 rounds discarded as burn‐in and a thinning of 20. Inference on all the parameters was obtained from the mean of the respective posterior distributions after discarding the burn‐in period. MCMC diagnostics were performed with the R package CODA (Plummer et al. 2006) (results not shown).

A recap of the effects of each model population combination is reported in Table 2.

**TABLE 2:** Recapitulation of effects in different models in the paper.

	Sire	CG	Fixed			Random		
			Mat. line	Sex	Pen	Litter	Genome	Microbiota
TE								
M	✓	✓	✓		✓			✓
G		✓	✓		✓		✓	
M + G		✓	✓		✓		✓	✓
NU								
M	✓	✓		✓		✓		✓
G		✓		✓		✓	✓	
M + G		✓		✓		✓	✓	✓

Abbreviations: CG, contemporary group; G, genomic effect; M + G, microbiota and genomic effects; M, microbiota effect; Mat. Line, maternal line; NU, nucleus population; TE, terminal population.

### 2.7 Predicted Measures

To assess the performance of microbial and genomic information in our prediction models, we calculated predictive abilities for various measures associated with the traits under investigation. These measures included:

*Phe*: These are the centre‐scaled raw phenotypes representing the unadjusted phenotypic measurements within each system. These measurements were not corrected for any systematic effects.

*PheAdg*: These phenotypes were adjusted for all fixed effects. In this case, the raw phenotypes were corrected for the effects of the contemporary group in both NU and TE populations. In addition, they were adjusted for the effects of maternal line and pen in the TE population and for sex in the NU population.

Both sets of phenotypic measures were considered to account for the potential of microbial information to capture some of the systematic variability implicitly included in the models. This approach allowed us to compare the prediction accuracy between these two different measures.

The predictive performance of each model/trait/time combination was evaluated through Pearson's correlation coefficient (*r*) of predicted and observed measures and root mean squared errors (RMSE).

## 3 Results

In this paper, we explored the combined effect of microbial and genomic information to predict growth performance in swine, specifically predicted performance across two systems connected by sires. These are representative of a typical North American system. As such, we focused on investigating the prediction of the opposite system (NU to TE and TE to NU) rather than concentrating on within‐population predictions. Nonetheless, we have reported results in the TE population in previous research (Maltecca et al. 2019).

### 3.1 Sire Stratified Scenario

**FIGURE 2:**
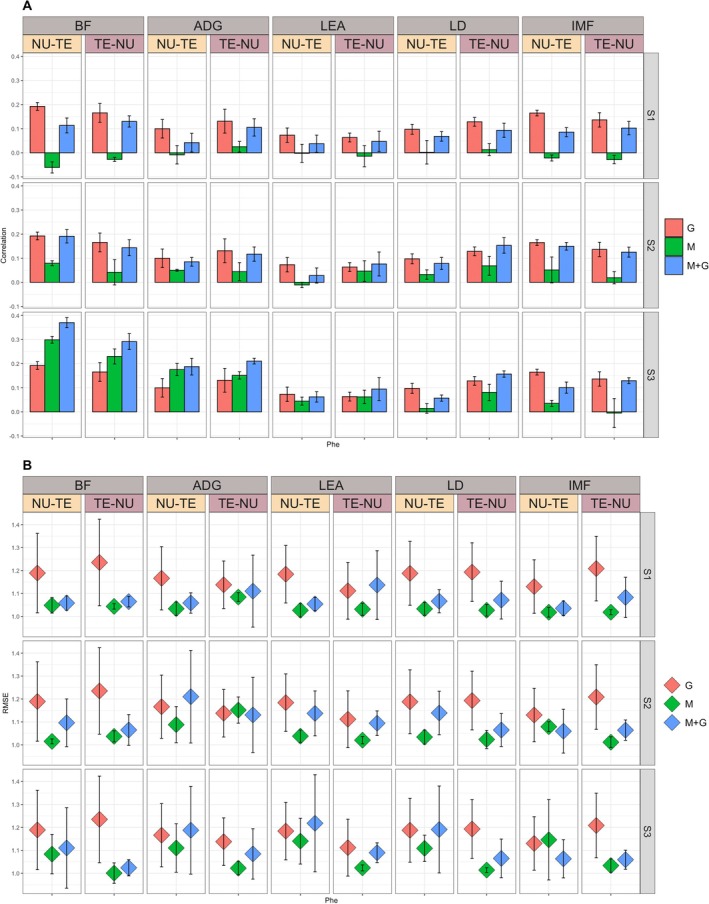
Prediction accuracy (Panel A) and RMSE (Panel B) (SD within parentheses) for raw phenotypes (Phe) for the sire stratified (SSt) scenario. ADG, average daily gain; BF, back fat; G, genome; IMF, intramuscular fat; LD, loin depth; LEA, loin area; M + G, microbiota + genome; M, microbiota; NU‐TE, training on nucleus and predicting on terminal; S1, microbial collection at weaning; S2, microbial collection at mid‐test; S3, microbial collection at off‐test; TE‐NU, training on terminal and predicting on nucleus. [Colour figure can be viewed at wileyonlinelibrary.com]

**FIGURE 3:**
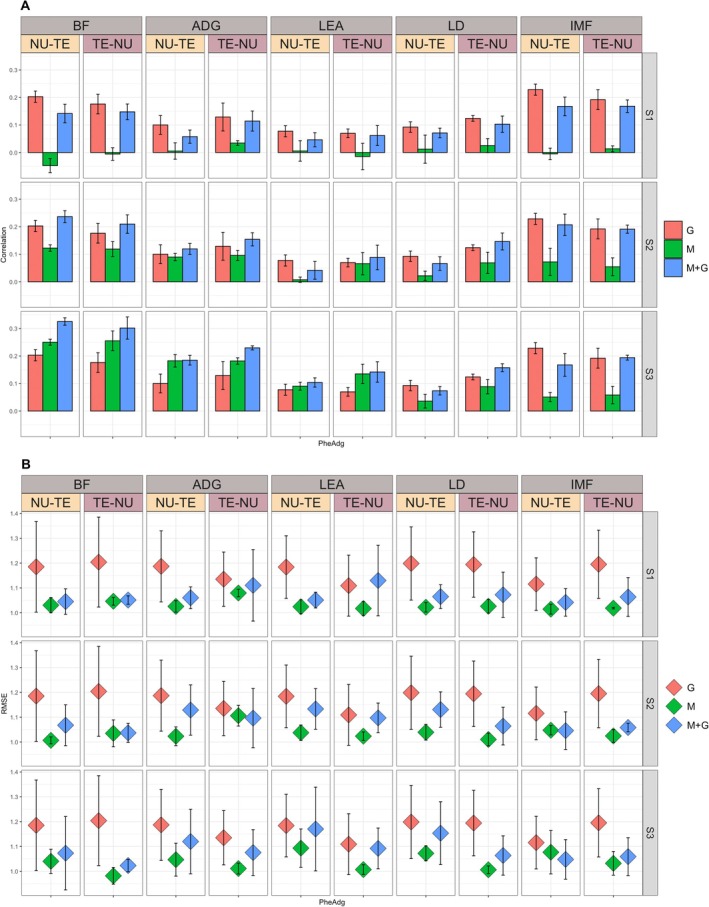
Prediction accuracy (Panel A) and RMSE (Panel B) (SD within parentheses) for adjusted phenotypes (PheAdg) sire stratified (SSt) scenario. ADG, average daily gain; BF, back fat; G, genome; IMF, intramuscular fat; LD, loin depth; LEA, loin area; M + G, microbiota + genome; M, microbiota; NU‐TE, training on nucleus and predicting on terminal; S1, microbial collection at weaning; S2, microbial collection at mid‐test; S3, microbial collection at off‐test; TE‐NU, training on terminal and predicting on nucleus. [Colour figure can be viewed at wileyonlinelibrary.com]

Results for the prediction of raw (Phe) and adjusted (PheAdg) phenotypes for the SSt scenario are reported in Figures 2A and 3A for correlations, while for RMSE in Figures 2B and 3B. We fitted models including alternatively genomic, microbiota, or a combination of both effects.

#### 3.1.1 Impact of Phenotype on Prediction

Including genomic information yielded prediction accuracies ranging from low for LEA (0.063 TE‐NU, 0.073 NU‐TE) to moderate for BF (0.192 NU‐TE, 0.165 TE‐NU) for Phe. For BF, LEA, and IMF predictions, accuracies were higher for NU‐TE than for TE‐NU (0.192 vs. 0.165; 0.073 vs. 0.063; 0.165 vs. 0.136, respectively), while for ADG and LD, the opposite was true (0.100 vs. 0.131; 0.097 vs. 0.129, respectively). As expected, accuracies for PheAdg were higher in magnitude and followed similar trends apart from IMF being the trait with the highest accuracies (0.229 vs. 0.192 for TE‐NU and NU‐TE, respectively).

#### 3.1.2 Impact of Microbiota Time Points

When including microbial information, accuracies were dependent on the time at collection. Prediction accuracy over time followed trends similar to ones found in previous works (Maltecca et al. 2019), and microbial information collected around weaning (S1) provided no predictive power for all traits. Pearson's correlations ranged from negative for BF (−0.06 vs. −0.02 for NU‐TE and TE‐NU, respectively) to essentially 0 for all other traits. Similarly, and in accordance with what was found by other studies, predictions were higher at later times (Verschuren et al. 2020). The highest accuracies were achieved for BF, where Pearson's correlations were 0.079 and 0.042, and 0.299 and 0.230 for S2 and S3, for NU‐TE and TE‐NU, respectively. Similarly, ADG accuracies were 0.050 and 0.044 and 0.176 and 0.151 for the same time points and scenario combinations. In this case, IMF was the trait for which Microbiota had the lowest predictive power at later time points with values of 0.05 and 0.02 for NU‐TE and TE‐NU at S2 and 0.035 and ~0 at S3 for the same scenarios. Models including only M generally had higher predictive power from NU‐TE for BF, ADG, and IMF. In contrast, the opposite was true for the remaining traits, albeit differences were minor apart from LD, where, at S3, accuracies in NU‐TE were lower by ~0.07 compared to TE‐NU.

#### 3.1.3 Impact of Genomic, Microbial, and Combined (G + M) on Prediction

When comparing the predictive power of genomic and microbial information across systems, M had higher prediction accuracies than G for BF (+0.107, +0.065; NU‐TE, TE‐NU) and ADG (+0.076, +0.02; NU‐TE, TE‐NU) at S3. Lower prediction accuracies were obtained for IMF (−0.130, −0.141; NU‐TE, TE‐NU), while substantially similar for LD and LEA. In all cases, G outperformed M at S1 and S2.

Adjusting phenotypes while including microbial information in predictions resulted in higher prediction accuracies for all traits in both NU‐TE and TE‐NU, ranging from +0.07 for ADG NU‐TE to +0.073 for LEA TE‐NU, except for BF where PheAdg had a lower correlation (−0.049) in NU‐TE, compared to Phe.

Including both genomic and microbial information in Phe prediction resulted in higher accuracies than M and G alone at S3 for BF (0.37 and 0.292 for NU‐TE and TE‐NU) and ADG (0.188 and 0.211 for NU‐TE and TE‐NU). Results for other traits differed for different scenarios, with M + G outperforming both G and M for LEA in TE‐NU by ~0.02 but not in NU‐TE.

Conversely, in most cases, M + G predictions at S3 for PheAdg were higher than G and M alone, except for IMF and LD in NU‐TE, in which G outperformed. Accuracies ranged from 0.325 (BF NU‐TE) to 0.074 (LD NU‐TE). Increases in accuracy compared to G ranged from approximately +0.12 for BF across both scenarios to about +0.02 for LEA in NU‐TE. In all cases, M + G had higher predictions than M alone.

In panel B of Figures 1 and 2, the RMSE of each model/scenario/time combination is reported. RMSE was similar across different combinations and generally slightly lower for M, followed by M + G and G.

### 3.2 Sire Family Left Cross Validation (SOut)

**FIGURE 4:**
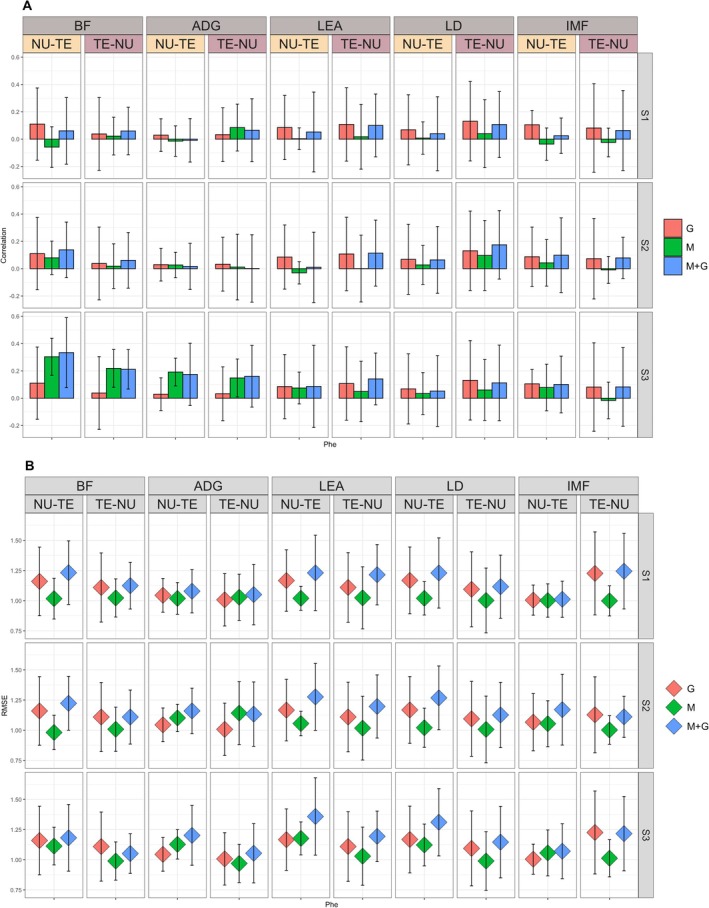
Prediction accuracy (Panel A) and RMSE (Panel B) (SD within parentheses) for raw phenotypes (Phe) for the sire Out (SOut) scenario. ADG, average daily gain; BF, back fat; G, genome; IMF, intramuscular fat; LD, loin depth; LEA, loin area; M + G, microbiota + genome; M, microbiota; NU‐TE, training on nucleus and predicting on terminal; S1, microbial collection at weaning; S2, microbial collection at mid‐test; S3, microbial collection at off‐test; TE‐NU, training on terminal and predicting on nucleus. [Colour figure can be viewed at wileyonlinelibrary.com]

**FIGURE 5:**
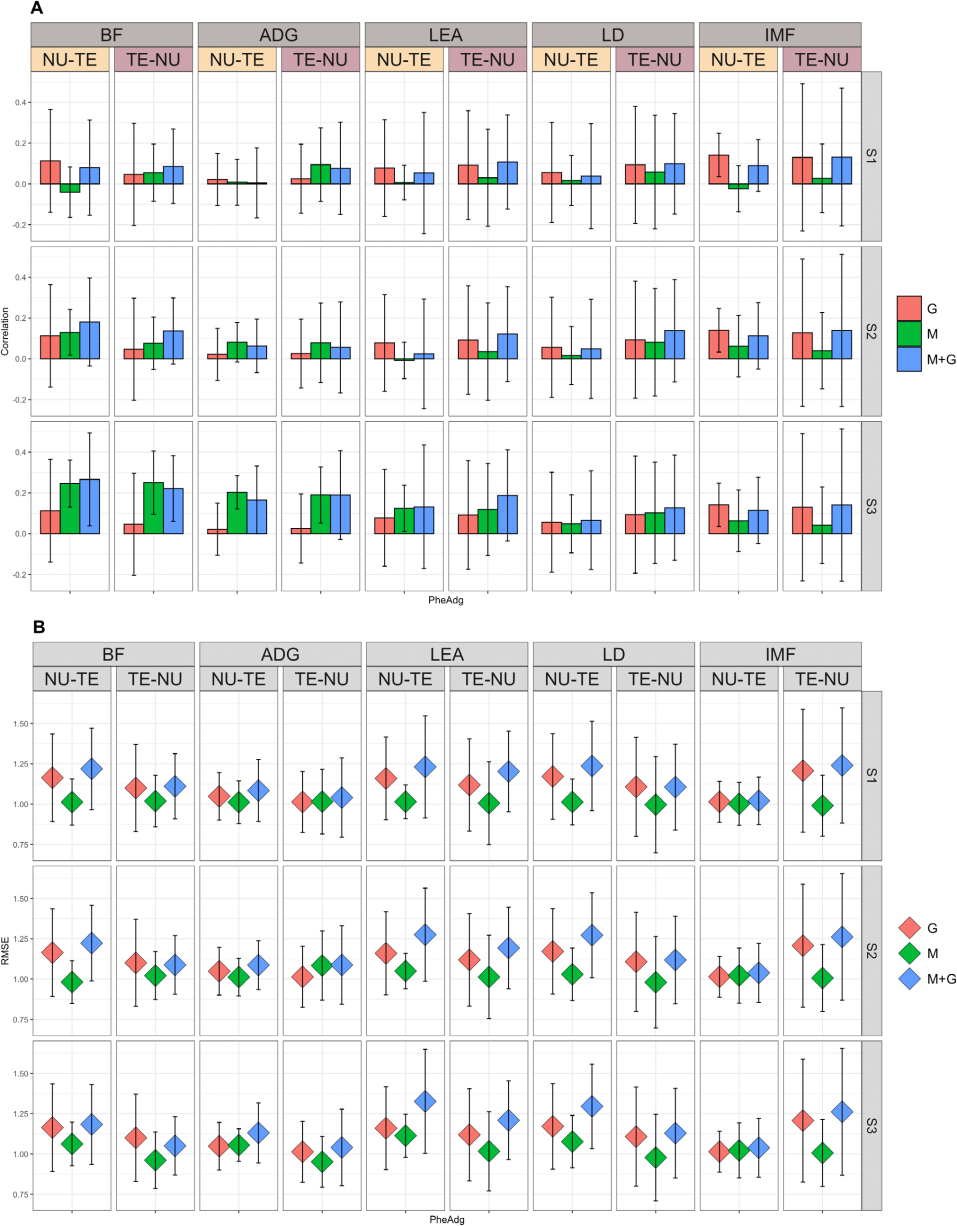
Prediction accuracy (Panel A) and RMSE (Panel B) (SD within parentheses) for adjusted phenotypes (PheAdg) sire out (SOut) scenario. ADG, average daily gain; BF, back fat; G, genome; IMF, intramuscular fat; LD, loin depth; LEA, loin area; M + G, microbiota + genome; M, microbiota; NU‐TE, training on nucleus and predicting on terminal; S1, microbial collection at weaning; S2, microbial collection at mid‐test; S3, microbial collection at off‐test; TE‐NU, training on terminal and predicting on nucleus. [Colour figure can be viewed at wileyonlinelibrary.com]

Results for predicting Phe and PheAdg for the SOut scenario are reported in Figures 4A,B and 5A, respectively.

Respect to SSt scenario, predictions based on genomic information only yielded relatively low prediction accuracies for Phe for all traits, ranging from approximately 0.03 for ADG both for NU‐TE and TE‐NU to approximately 0.13 for LD in TE‐NU (~0.11 NU‐TE) as reported in Figure 4A. When predicting PheAdg, as expected, accuracies were higher for most traits except for ADG, which remained poorly predicted (~0.03 for both NU‐TE and TE‐NU). In most cases, prediction accuracies were consistent when using NU or TE as the training population, apart from BF, where predictions from NU‐TE were 0.11 versus 0.05 when predicting from TE‐NU. In all cases, there was a much larger variability than the SSt (average SD across traits 0.232 for SOut vs. 0.063 for SSt) scenario, indicating that some families were predicted better than others. This could be due to differences in genomic resemblance between the training population and the testing family or since the number of individuals in training and testing varied slightly depending on the numerosity of the family excluded. In all cases, predictions were lower than in scenario SSt. This should be expected given that cross‐validation was performed, excluding training individuals from the same sire family as the ones in predictions.

Accuracies of prediction based on microbial information only, just as in the SSt scenario, were dependent on the sampling time. When considering Phe at S1, microbiota abundance had no predictive power, with an average accuracy of essentially 0 (0.004) across all traits and for both NU‐TE and TE‐NU. At S2, accuracies were still low and ranged from essentially 0 for LEA in both TE‐NU and NU‐TE to around 0.08 for BF in NU‐TE (0.02 in TE‐NU). Again, standard deviations were large in all cases and included 0 for all traits. At S3, in contrast, prediction accuracies were moderate for ADG (0.19 NU‐TE and 0.15 TE‐NU) and BF (0.30 NU‐TE and 0.22 TE‐NU), while they were higher than S2 but still low for LEA (0.07 NU‐TE and 0.05 TE‐NU), LD (0.03 NU‐TE and 0.06 TE‐NU), and IMF (0.08 NU‐TE and ~0 TE‐NU). In the case of ADG (+0.16 NU‐TE and +0.11 TE‐NU) and BF (+0.19 NU‐TE and +0.18 TE‐NU), microbiota prediction accuracies were substantially larger than the one obtained with G.

The inclusion of both M and G did not significantly improve prediction accuracy compared to M, except for BF in NU‐TE (+0.03), LEA in TE‐NU (+0.09), and LD in TE‐NU (+0.05). Concerning G, M + G was not better than G for all traits for which M alone had poor predictive power, as expected.

In panel B of Figures 4B and 5B, the RMSE of each model/scenario/time combination is reported. Like SSt, RMSE was comparable across different combinations and generally slightly lower for M, followed by G and M + G.

Results from the permutation analysis SNull are reported in Table 3. Only results for which both accuracies of SSt and SOut exceeded the 95% percentile of the empirical null distribution are reported, while all the complete table is available in Table S3. For all permutation models, the prediction accuracy was essentially 0 (mean 0.006), significantly lower than the predictions obtained for SSt and SOut, confirming that both genomic information and microbiota abundance were able to predict phenotypes in the NU and TE populations. When comparing results across the three SSt, SOut, and SNull, we can see that for ADG at all time points, models including M or M + G exceeded the 95% percentile of the empirical null distribution, while in most cases, G did not for the scenario SOut. For BF, all models G, M, and G + M exceeded the 95% threshold apart from S1, where only G was a significantly better predictor than the permutated model. M alone was never better than the permutated model for the remaining traits in both SSt and SOut, except for LD TE‐NU at S3.

**TABLE 3:** Accuracy of prediction in permutation analysis.^a^

Scenario	Trait	Time	Model	Acc‐SOut	Acc‐SSst	Acc‐SNull	Acc‐SNull90%	Acc‐SNull95%
TE‐NU	ADG	S1	M + G	0.065	0.105	0.006	0.034	0.041
NU‐TE	ADG	S2	M	0.027	0.050	0.005	0.047	0.059
TE‐NU	ADG	S2	M	0.012	0.044	0.005	0.032	0.039
NU‐TE	ADG	S3	M	0.192	0.176	0.007	0.025	0.029
TE‐NU	ADG	S3	M	0.148	0.151	0.006	0.035	0.043
NU‐TE	ADG	S3	M + G	0.174	0.188	0.007	0.029	0.036
TE‐NU	ADG	S3	M + G	0.161	0.211	0.006	0.034	0.042
NU‐TE	BF	S1	G	0.110	0.192	0.005	0.039	0.046
NU‐TE	BF	S1	M + G	0.061	0.113	0.007	0.044	0.058
TE‐NU	BF	S1	M + G	0.060	0.130	0.007	0.044	0.054
NU‐TE	BF	S2	G	0.110	0.192	0.005	0.03	0.039
NU‐TE	BF	S2	M	0.079	0.079	0.008	0.045	0.057
NU‐TE	BF	S2	M + G	0.138	0.191	0.006	0.045	0.058
TE‐NU	BF	S2	M + G	0.061	0.144	0.007	0.045	0.055
NU‐TE	BF	S3	G	0.110	0.192	0.007	0.033	0.04
NU‐TE	BF	S3	M	0.304	0.299	0.007	0.031	0.036
TE‐NU	BF	S3	M	0.218	0.230	0.007	0.047	0.057
NU‐TE	BF	S3	M + G	0.334	0.370	0.008	0.049	0.059
TE‐NU	BF	S3	M + G	0.212	0.292	0.007	0.031	0.036
NU‐TE	IMF	S1	G	0.105	0.165	0.007	0.032	0.038
TE‐NU	IMF	S1	G	0.082	0.136	0.006	0.041	0.049
TE‐NU	IMF	S1	M + G	0.063	0.102	0.007	0.037	0.045
NU‐TE	IMF	S2	G	0.087	0.165	0.006	0.034	0.041
TE‐NU	IMF	S2	G	0.073	0.136	0.008	0.039	0.05
NU‐TE	IMF	S2	M + G	0.099	0.149	0.006	0.033	0.042
TE‐NU	IMF	S2	M + G	0.079	0.125	0.006	0.041	0.05
NU‐TE	IMF	S3	G	0.105	0.165	0.005	0.033	0.041
TE‐NU	IMF	S3	G	0.082	0.136	0.006	0.041	0.05
NU‐TE	IMF	S3	M	0.079	0.035	0.006	0.03	0.036
NU‐TE	IMF	S3	M + G	0.101	0.101	0.006	0.026	0.03
TE‐NU	IMF	S3	M + G	0.083	0.129	0.005	0.043	0.053
NU‐TE	LD	S1	G	0.068	0.097	0.007	0.026	0.031
TE‐NU	LD	S1	G	0.131	0.129	0.006	0.027	0.033
TE‐NU	LD	S1	M + G	0.107	0.093	0.007	0.033	0.042
NU‐TE	LD	S2	G	0.068	0.097	0.006	0.025	0.029
TE‐NU	LD	S2	G	0.131	0.129	0.004	0.051	0.062
TE‐NU	LD	S2	M	0.097	0.069	0.005	0.047	0.058
NU‐TE	LD	S2	M + G	0.064	0.078	0.005	0.044	0.052
TE‐NU	LD	S2	M + G	0.175	0.153	0.005	0.027	0.032
NU‐TE	LD	S3	G	0.068	0.097	0.005	0.029	0.034
TE‐NU	LD	S3	G	0.131	0.129	0.006	0.049	0.064
TE‐NU	LD	S3	M	0.060	0.081	0.009	0.051	0.063
NU‐TE	LD	S3	M + G	0.052	0.057	0.007	0.044	0.054
TE‐NU	LD	S3	M + G	0.112	0.157	0.005	0.042	0.053
NU‐TE	LEA	S1	G	0.085	0.073	0.007	0.050	0.064
TE‐NU	LEA	S1	G	0.108	0.063	0.006	0.032	0.039
NU‐TE	LEA	S1	M + G	0.052	0.038	0.007	0.027	0.033
TE‐NU	LEA	S1	M + G	0.100	0.047	0.006	0.028	0.034
NU‐TE	LEA	S2	G	0.085	0.073	0.006	0.031	0.038
TE‐NU	LEA	S2	G	0.108	0.063	0.006	0.025	0.029
TE‐NU	LEA	S2	M + G	0.114	0.076	0.006	0.025	0.029
NU‐TE	LEA	S3	G	0.085	0.073	0.007	0.032	0.039
TE‐NU	LEA	S3	G	0.108	0.063	0.006	0.033	0.04
NU‐TE	LEA	S3	M + G	0.086	0.062	0.006	0.024	0.028
TE‐NU	LEA	S3	M + G	0.141	0.095	0.006	0.025	0.030

Abbreviations: Acc‐SNull, accuracy of prediction for phenotype (Phe) for the permutation (SNull) cross‐validation; Acc‐SNull90%, 90th percentile of accuracy of prediction for phenotype (Phe) for the permutation (SNull) cross‐validation; Acc‐SNull95%, 95th percentile of accuracy of prediction for phenotype (Phe) for the permutation (SNull) cross‐validation; Acc‐SOut, accuracy of prediction for phenotype (Phe) for the sire out (SOut) cross‐validation; Acc‐SSt, accuracy of prediction for phenotype (Phe) for the sire stratified (SSt) cross‐validation; ADG, average daily gain; BF, back fat; G, genome; IMF, intramuscular fat; LD, loin depth; LEA, loin area; M + G, microbiota + genome; M, microbiota; NU‐TE, training on nucleus and predicting on terminal; S1, microbial collection at weaning; S2, microbial collection at mid‐test; S3, microbial collection at off‐test; TE‐NU, training on terminal and predicting on nucleus.

^a^ Only results for which both accuracies of SSt and SOut exceeded the 95% percentile of the empirical null distribution.

Table 4 summarises all results across all scenarios. BF achieved the highest prediction accuracies, especially for PheAdg using combined genomic and microbiota data (G + M) at S3, reaching 0.370 (NU‐TE) and 0.292 (TE‐NU). In contrast, LEA consistently had the lowest accuracies, particularly in the SOut scenario. Adjusting phenotypes (PheAdg) generally improved prediction accuracies across traits, except for BF in NU‐TE, where raw phenotypes performed slightly better. G + M models outperformed both G and, notably, M alone. Regarding microbiota time points, S1 (weaning) offered no predictive power, while S3 (off‐test) performed best. Predictions were more accurate and stable in the SSt (sire‐stratified) scenario than SOut (sire‐out). Slight advantages favoured NU‐TE over TE‐NU for BF, IMF, and LEA, whereas TE‐NU performed slightly better for ADG and LD. RMSE values were similar across models, with marginally lower errors for M‐only predictions.

**TABLE 4:** This table provides a comprehensive overview of prediction accuracies and root mean square errors (RMSE) for different models (genomic, microbiota, and combined) across sire‐stratified (SSt) and sire‐out (SOut) scenarios.

Variable on prediction	Best	Worst
Phenotype (trait)	BF (back fat)—highest accuracies across SSt & SOut	LEA (loin eye area)—lowest accuracies especially in SOut
Adjusted phenotype	Adjusted phenotype	Raw phenotype
Model	G + M (genomic + microbiota)—consistently highest prediction accuracies at S3	M only (microbiota)—negligible predictive power at S1
Time point	S3 (off‐test)—microbiota strongest predictive power	S1 (weaning)—microbiota provided no predictive power
Scenario	SSt (sire‐stratified)—higher, more stable accuracies (avg SD 0.063)	SOut (sire‐out)—lower accuracies, high variability (avg SD 0.232)
Prediction direction	NU‐TE—slightly better for BF, IMF, LEA in most cases	TE‐NU—slightly better for ADG, LD in some cases
Overall accuracy	BF (Phe, SSt, G + M, S3)	ADG (Phe, SOut, G only)

*Note:* It includes results for raw and adjusted phenotypes, microbial sampling time points, and training/testing population combinations (NU‐TE and TE‐NU). The summary highlights key patterns in model performance, phenotype predictability, and the impact of microbiota sampling times.

## 4 Discussion

In the current paper, we evaluated the power of faecal microbial composition and genomic information to foretell growth performance across farming systems. We took advantage of an experimental design where the microbial composition of purebred or terminal crosses progeny of 28 Duroc sires was collected at three time points during the performance test. We then used this information to investigate how OTU counts and genomic information could be used to predict phenotypes in the nucleus or terminal populations. To our knowledge, this is the first of such investigations.

In our analysis, prediction accuracy depended on the sampling time, and microbiota abundance collected closer to phenotypes was better predictors of phenotypes for all traits and in both systems. These results are in accordance with previous studies from our and other groups (Verschuren et al. 2020; Ridaura et al. 2013). In our research, prediction accuracy from NU to TE was generally higher than the opposite for BF and ADG, while the opposite was true for LD IMF and LEA.

These findings align with previous studies (Camarinha‐Silva et al. 2017; Plummer et al. 2006), which demonstrated that the gut microbiota explains a substantial proportion (often exceeding 20%–30%) of phenotypic variance in growth and carcass quality traits. This strong association highlights the microbiota's critical role in shaping these economically important characteristics. Specifically, in pigs, variations in gut microbial composition have been closely linked to differences in growth rate and backfat thickness. Such connections arise because specific microbial communities modulate host metabolism, directly influencing energy partitioning between muscle growth and fat deposition (Maltecca et al. 2019; Godinho et al. 2018; Tang et al. 2020; Hu et al. 2025; Cao et al. 2022).

Possible reasons for this could include greater environmental variability in commercial settings, which may lead to a broader microbial representation in the TE population, as well as differences in sample size between the training populations. Prediction accuracies were greater than zero for all traits and were substantial for most, particularly for fat deposition and growth. This was observed when predicting from NU to TE and vice versa. Additionally, the discrepancy in prediction accuracy between the NU and TE populations might also be explained by biological differences between the two phenotypes, which differed considerably.

Since the two systems are separated, and the only common conditions shared were sire and microbiota, we find these results interesting. High correlations across systems are even more relevant, considering that the traits measured in the two populations were not the same (Khanal et al. 2020).

When predicting adjusted phenotypes, results are generally similar, with higher correlations for adjusted phenotypes in most cases, except BF for NU‐TE at S3, for which raw phenotypes were better predicted. When predicting from nucleus to terminal, correcting for all systematic effects lowered the predictive ability of microbiota information by roughly 4%, suggesting that microbiota information recapitulates some systematic variability captured in the models. It is hard to pinpoint precisely what since the sire is the only common systematic effect between the two systems. Still, microbiota information is probably collinear with sire or systematic environmental variation. Research in this area should be expanded to explain these contrasting results better. With a more granular description of environmental variation, it would be possible to describe discrepancies and similarities between the systems that cannot be captured by the current design.

In our study, for BF, ADG, and LEA, Microbiota collected at S3 was a better predictor than genomic information of PheAdg in both NU‐TE and TE‐NU. On average and across systems, M predictions were ~0.06 more accurate than G for BF, ~0.07 for ADG, and ~0.04 for LEA. Similarly, when considering Phe, M was a better predictor than G for BF (+~0.09) and ADG, while for the latter, margins were smaller for TE‐NU (+0.02). Again, this is not surprising considering that Microbiota should be able to recapitulate a larger proportion of phenotypic variability than just the genomic, and previous studies have highlighted (Déru et al. 2021) the predictive power of microbial composition on growth and efficiency parameters in swine.

Nonetheless, this is the first attempt to use microbial information to predict systems that are largely non‐overlapping in space, time, management, and systematic environmental conditions, and further work would be needed to validate these results. In most cases including S3, M, and G in the model increased prediction accuracies for both Phe and PheAdg. The advantage was again more considerable for fat deposition and ADG, which was true for both TE‐NU and NU‐TE. While there was an advantage in including M + G, the increase in accuracy was not proportional to the sum of the two effects, suggesting partial collinearity between G and M, as previously found in similar studies (Cao et al. 2022; Déru et al. 2021). Previous work from our and other groups (Martínez‐Álvaro et al. 2020; Bergamaschi, Maltecca, Schillebeeckx, et al. 2020) has provided some evidence of a genetic basis for microbial composition. However, the interplay between G and M is complex, and few studies have so far tried to establish the causal relationship between the two (Christensen et al. 2021; Tiezzi et al. 2021). Microbiota resemblance between individuals might be the result of genomic similarity among individuals. Our current design could not disentangle whether that is due to independent causes or through a direct effect of the host on its microbial makeup.

Comparing the SSt and SOut scenarios, we saw that predictions in the first were generally higher than in the second. This was true for all combinations of information (genomic and microbiota). The SSt scenario was about 0.05 more accurate than SOut (averaged across time, source of information, and trait). Interestingly, for BF and ADG, M was the best predictor, followed by M + G models at time points S2 and S3. This was not the case for LD LEA and IMF. In SOut, information from relatives of the validation sets was removed in training. This resulted in G's poor predictive ability, particularly in the TE‐NU scenario. In this case, though, even for M and M + G, prediction accuracies were lower for SOut than SSt. Reasons for this are various and might include the removal in SOut of some shared covariance due to the same sire pens, the different sizes of training and validation due to family size difference, and again, the removal of relatives in training owing to the partial genetic base of microbial composition. In most cases, whether in the SSt or SOut, prediction exceeded the 90th percentile of the empirical null, suggesting that there is actual signal in microbial composition to predict growth performance across systems.

While it has been speculated that microbial information could serve as a biomarker of individuals' physiological status, this aspect was not explicitly considered in the current study, and additional investigation is warranted (Ma et al. 2023). Within the scope of the current paper, we identified how microbiota composition can predict the performance of different (albeit genetically connected) systems.

Ideally, as suggested by our and other studies, if microbial composition has both a genomic component but also serves as a measure of environmental covariance, it would be possible to explicitly include the genetic effects of microbial composition either as an effect in prediction models with co‐variance with the additive genetic effect or directly as a correlated trait in multivariate models for the heritable microbial features. Features that contribute only to environmental covariance could be, in turn, effectively modelled as correlated residuals as in multi‐environmental models. González‐Recio et al. (2023) outline some of these models' possible advantages and disadvantages. As mentioned, it is possible to jointly model both genomic and microbiome effects using their respective variance–covariance matrices. Additionally, estimating the covariance between these two effects to assess their correlation can provide deeper insights into host genome–microbiome interactions for phenotype prediction. We are going to explore this approach further in ongoing research.

It is essential to recognise that the value of microbial information will be tightly linked to the amount of information it carries within this context. Collecting phenotypes for a trait in a commercial system might be far cheaper than measuring microbial composition. Nonetheless, and as already suggested, microbial information could, at least in theory, capture a broader amount of signal. The current study is limited in this regard by providing only an incomplete indication of the potential predictive value of microbiota without an indication of overall economic feasibility. Future studies should focus on a complete characterisation of environmental variability across different production systems and collecting extra information on individuals' physiological status.

While the study was not conducted to investigate a genetic component of microbial composition, a family effect appears evident and reinforces results from other studies (Crespo‐Piazuelo et al. 2019). Nonetheless, larger datasets and more appropriate analysis methods should be employed to investigate this aspect further. If a host component is confirmed, the microbial composition could be included in selection goals as an explicit selection criterion, as suggested by Weishaar et al. (2020).

## 5 Conclusions

We investigated the effectiveness of microbial composition in predicting phenotypes across two standard swine production systems. We found that microbial information can predict performance for most growth and carcass composition traits, particularly for growth and fat deposition. We found that microbial predicted values are relatively stable across systems. We found that both microbial and genomic information contribute to predicting phenotypes, but only in a few cases including both Microbiota and genomic information resulting in higher prediction accuracies than including one of the two. We found that microbial information was a better predictor for fat deposition and growth than genomics. Results from the current study could help design data collection to increase information flow from diverse production systems used for selecting individuals in swine.

## Author Contributions

C.M. and F.T. conceived the study and designed the experiment. C.M. performed the statistical analysis. C.M., F.T., J.J., E.M., M.C.F., and R.B. contributed to interpreting the results. C.M. and F.T. wrote the first draft of the manuscript. All authors contributed to the manuscript review and editing. All authors read and approved the final manuscript.

## Ethics Statement

The authors have nothing to report.

## Consent

The authors have nothing to report.

## Conflicts of Interest

The authors declare no conflicts of interest.

## Supporting information


**Table S1:** jbg70014‐sup‐0001‐TablesS1‐S3.docx.
**Table S2:** jbg70014‐sup‐0001‐TablesS1‐S3.docx.
**Table S3:** jbg70014‐sup‐0001‐TablesS1‐S3.docx.

## Data Availability

The data that support the findings of this study are available on request from the corresponding author. The data are not publicly available due to privacy or ethical restrictions.
