# Supplementary material for: Microbial and Genomic Information Synergistically Contribute to Predicting Swine Performance Across Production Systems
Source: J Anim Breed Genet. 2025 Sep 24;143(2):256–72. doi: 10.1111/jbg.70014 (PMC12887147; doi:10.1111/jbg.70014)
Supplement: Supplementary file 1 — Table S1: jbg70014‐sup‐0001‐TablesS1‐S3.docx. Table S2: jbg70014‐sup‐0001‐TablesS1‐S3.docx. Table S3: jbg70014‐sup‐0001‐TablesS1‐S3.docx. [file JBG-143-256-s001.docx]

|  | OADG | OBF | OLEA | OLD | OIMF |
| --- | --- | --- | --- | --- | --- |
| MADG | **0.464**  (0.137; 0.778) | 0.456  (0.202; 0.714) | 0.788  (0.634; 0.939) | 0.784  (0.612; 0.903) | -0.112  (-0.490; 0.283) |
| MBF | -0.220  (-0.623; 0.152) | **0.423**  (0.101; 0.649) | -0.019  (-0.397; 0.378) | 0.224  (-0.145; 0.548) | 0.293  (-0.072; 0.692) |
| MLEA | 0.277  (-0.082; 0.586) | -0.312  (-0.582; 0.013) | **0.665**  (0.472; 0.853) | 0.546  (0.254; 0.795) | -0.163  (-0.552; 0.222) |
| MLD | 0.277  (-0.082; 0.586) | -0.312  (-0.582; 0.013) | 0.665  (0.472; 0.853) | **0.546**  (0.254; 0.795) | -0.163  (-0.552; 0.222) |
| MIMF | -0.111  (-0.420; 0.286) | 0.548  (0.298; 0.780) | -0.040  (-0.357; 0.355) | 0.043  (-0.334; 0.386) | **0.946**  (0.909; 0.978) |

**Supplementary Table 1:** Genetic Correlations between traits measured in the terminal (TE) population [MADG=Market average daily gain; MBF= Market backfat; MLEA= Market loin area; MLD= Market loin depth; MIMF= Market intramuscular fat] and in the nucleus (NU) population [OADG=Offtest average daily gain; OBF= Offtest backfat; OLEA= Offtest loin area; OLD= Offtest loin depth; OIMF= Offtest intramuscular fat].

| **Cross Validation** | **Phenotype**  **Correction** | **Type of Prediction** | **Metrics of Prediction** | **Time point of Microbiota** | **Type of Figure** | **Figure in the Manuscript** |
| --- | --- | --- | --- | --- | --- | --- |
| SSt | Phe | NU-TE | Corr | S1 | M | Figure 2  (Pannel A) |
|  |  |  |  |  | G |  |
|  |  |  |  |  | G+M |  |
|  |  |  |  | S2 | M |  |
|  |  |  |  |  | G |  |
|  |  |  |  |  | G+M |  |
|  |  |  |  | S3 | M |  |
|  |  |  |  |  | G |  |
|  |  |  |  |  | G+M |  |
|  |  |  | RMSE | S1 | M | Figure 2  (Pannel B) |
|  |  |  |  |  | G |  |
|  |  |  |  |  | G+M |  |
|  |  |  |  | S2 | M |  |
|  |  |  |  |  | G |  |
|  |  |  |  |  | G+M |  |
|  |  |  |  | S3 | M |  |
|  |  |  |  |  | G |  |
|  |  |  |  |  | G+M |  |
|  |  | TE-NU | Corr | S1 | M | Figure 2  (Pannel A) |
|  |  |  |  |  | G |  |
|  |  |  |  |  | G+M |  |
|  |  |  |  | S2 | M |  |
|  |  |  |  |  | G |  |
|  |  |  |  |  | G+M |  |
|  |  |  |  | S3 | M |  |
|  |  |  |  |  | G |  |
|  |  |  |  |  | G+M |  |
|  |  |  | RMSE | S1 | M | Figure 2  (Pannel B) |
|  |  |  |  |  | G |  |
|  |  |  |  |  | G+M |  |
|  |  |  |  | S2 | M |  |
|  |  |  |  |  | G |  |
|  |  |  |  |  | G+M |  |
|  |  |  |  | S3 | M |  |
|  |  |  |  |  | G |  |
|  |  |  |  |  | G+M |  |
|  | PheAdg | NU-TE | Corr | S1 | M | Figure 3  (Pannel A) |
|  |  |  |  |  | G |  |
|  |  |  |  |  | G+M |  |
|  |  |  |  | S2 | M |  |
|  |  |  |  |  | G |  |
|  |  |  |  |  | G+M |  |
|  |  |  |  | S3 | M |  |
|  |  |  |  |  | G |  |
|  |  |  |  |  | G+M |  |
|  |  |  | RMSE | S1 | M | Figure 3  (Pannel B) |
|  |  |  |  |  | G |  |
|  |  |  |  |  | G+M |  |
|  |  |  |  | S2 | M |  |
|  |  |  |  |  | G |  |
|  |  |  |  |  | G+M |  |
|  |  |  |  | S3 | M |  |
|  |  |  |  |  | G |  |
|  |  |  |  |  | G+M |  |
|  |  | TE-NU | Corr | S1 | M | Figure 3  (Pannel A) |
|  |  |  |  |  | G |  |
|  |  |  |  |  | G+M |  |
|  |  |  |  | S2 | M |  |
|  |  |  |  |  | G |  |
|  |  |  |  |  | G+M |  |
|  |  |  |  | S3 | M |  |
|  |  |  |  |  | G |  |
|  |  |  |  |  | G+M |  |
|  |  |  | RMSE | S1 | M | Figure3  (Pannel B) |
|  |  |  |  |  | G |  |
|  |  |  |  |  | G+M |  |
|  |  |  |  | S2 | M |  |
|  |  |  |  |  | G |  |
|  |  |  |  |  | G+M |  |
|  |  |  |  | S3 | M |  |
|  |  |  |  |  | G |  |
|  |  |  |  |  | G+M |  |
| **SOut** | Phe | NU-TE | Corr | S1 | M | Figure 4  (Pannel A) |
|  |  |  |  |  | G |  |
|  |  |  |  |  | G+M |  |
|  |  |  |  | S2 | M |  |
|  |  |  |  |  | G |  |
|  |  |  |  |  | G+M |  |
|  |  |  |  | S3 | M |  |
|  |  |  |  |  | G |  |
|  |  |  |  |  | G+M |  |
|  |  |  | RMSE | S1 | M | Figure 4  (Pannel B) |
|  |  |  |  |  | G |  |
|  |  |  |  |  | G+M |  |
|  |  |  |  | S2 | M |  |
|  |  |  |  |  | G |  |
|  |  |  |  |  | G+M |  |
|  |  |  |  | S3 | M |  |
|  |  |  |  |  | G |  |
|  |  |  |  |  | G+M |  |
|  |  | TE-NU | Corr | S1 | M | Figure 4  (Pannel A) |
|  |  |  |  |  | G |  |
|  |  |  |  |  | G+M |  |
|  |  |  |  | S2 | M |  |
|  |  |  |  |  | G |  |
|  |  |  |  |  | G+M |  |
|  |  |  |  | S3 | M |  |
|  |  |  |  |  | G |  |
|  |  |  |  |  | G+M |  |
|  |  |  | RMSE | S1 | M | Figure 4  (Pannel B) |
|  |  |  |  |  | G |  |
|  |  |  |  |  | G+M |  |
|  |  |  |  | S2 | M |  |
|  |  |  |  |  | G |  |
|  |  |  |  |  | G+M |  |
|  |  |  |  | S3 | M |  |
|  |  |  |  |  | G |  |
|  |  |  |  |  | G+M |  |
|  | PheAdg | NU-TE | Corr | S1 | M | Figure 5  (Pannel A) |
|  |  |  |  |  | G |  |
|  |  |  |  |  | G+M |  |
|  |  |  |  | S2 | M |  |
|  |  |  |  |  | G |  |
|  |  |  |  |  | G+M |  |
|  |  |  |  | S3 | M |  |
|  |  |  |  |  | G |  |
|  |  |  |  |  | G+M |  |
|  |  |  | RMSE | S1 | M | Figure 5  (Pannel B) |
|  |  |  |  |  | G |  |
|  |  |  |  |  | G+M |  |
|  |  |  |  | S2 | M |  |
|  |  |  |  |  | G |  |
|  |  |  |  |  | G+M |  |
|  |  |  |  | S3 | M |  |
|  |  |  |  |  | G |  |
|  |  |  |  |  | G+M |  |
|  |  | TE-NU | Corr | S1 | M | Figure 5  (Pannel A) |
|  |  |  |  |  | G |  |
|  |  |  |  |  | G+M |  |
|  |  |  |  | S2 | M |  |
|  |  |  |  |  | G |  |
|  |  |  |  |  | G+M |  |
|  |  |  |  | S3 | M |  |
|  |  |  |  |  | G |  |
|  |  |  |  |  | G+M |  |
|  |  |  | RMSE | S1 | M | Figure5  (Pannel B) |
|  |  |  |  |  | G |  |
|  |  |  |  |  | G+M |  |
|  |  |  |  | S2 | M |  |
|  |  |  |  |  | G |  |
|  |  |  |  |  | G+M |  |
|  |  |  |  | S3 | M |  |
|  |  |  |  |  | G |  |
|  |  |  |  |  | G+M |  |

**Supplementary Table 3**. Summary of all analysis combinations and the figures where results are presented.

NU-TE= Training on Nucleus and predicting on Terminal. TE-NU= Training on Terminal and predicting on Nucleus.

S1= microbial collection at weaning. S2= microbial collection at mid-test. S3=microbial collection at off-test

M=Microbiome. G=Genome. M+G= Microbiome + Genome

Corr=Person Correlation

RMSE=Root Mean Squared Error

Phen=Phenotype

PhenAdg=Adjusted Phenotype

| Scenario | Trait | Time | Model | Acc-Sout | Acc-Sst | Acc-Snull | Acc-Snull90% | Acc-Snull95% |
| --- | --- | --- | --- | --- | --- | --- | --- | --- |
| NU-TE | ADG | S1 | G | 0.029 | 0.100 | 0.005 | 0.044 | 0.056 |
| TE-NU | ADG | S1 | G | 0.033 | 0.131 | 0.007 | 0.05 | 0.061 |
| NU-TE | ADG | S1 | M | -0.014 | -0.008 | 0.007 | 0.031 | 0.039 |
| TE-NU | ADG | S1 | M | 0.085 | 0.025 | 0.007 | 0.044 | 0.054 |
| NU-TE | ADG | S1 | M+G | -0.009 | 0.042 | 0.006 | 0.047 | 0.058 |
| TE-NU | ADG | S1 | M+G | 0.065 | 0.105 | 0.006 | 0.034 | 0.041 |
| NU-TE | ADG | S2 | G | 0.029 | 0.100 | 0.006 | 0.029 | 0.036 |
| TE-NU | ADG | S2 | G | 0.033 | 0.131 | 0.005 | 0.036 | 0.044 |
| NU-TE | ADG | S2 | M | 0.027 | 0.050 | 0.005 | 0.047 | 0.059 |
| TE-NU | ADG | S2 | M | 0.012 | 0.044 | 0.005 | 0.032 | 0.039 |
| NU-TE | ADG | S2 | M+G | 0.017 | 0.085 | 0.006 | 0.031 | 0.036 |
| TE-NU | ADG | S2 | M+G | 0.002 | 0.117 | 0.007 | 0.031 | 0.038 |
| NU-TE | ADG | S3 | G | 0.029 | 0.100 | 0.007 | 0.046 | 0.062 |
| TE-NU | ADG | S3 | G | 0.033 | 0.131 | 0.006 | 0.05 | 0.062 |
| NU-TE | ADG | S3 | M | 0.192 | 0.176 | 0.007 | 0.025 | 0.029 |
| TE-NU | ADG | S3 | M | 0.148 | 0.151 | 0.006 | 0.035 | 0.043 |
| NU-TE | ADG | S3 | M+G | 0.174 | 0.188 | 0.007 | 0.029 | 0.036 |
| TE-NU | ADG | S3 | M+G | 0.161 | 0.211 | 0.006 | 0.034 | 0.042 |
| NU-TE | BF | S1 | G | 0.110 | 0.192 | 0.005 | 0.039 | 0.046 |
| TE-NU | BF | S1 | G | 0.038 | 0.165 | 0.004 | 0.052 | 0.06 |
| NU-TE | BF | S1 | M | -0.058 | -0.061 | 0.006 | 0.03 | 0.038 |
| TE-NU | BF | S1 | M | 0.022 | -0.027 | 0.005 | 0.042 | 0.053 |
| NU-TE | BF | S1 | M+G | 0.061 | 0.113 | 0.007 | 0.044 | 0.058 |
| TE-NU | BF | S1 | M+G | 0.060 | 0.130 | 0.007 | 0.044 | 0.054 |
| NU-TE | BF | S2 | G | 0.110 | 0.192 | 0.005 | 0.03 | 0.039 |
| TE-NU | BF | S2 | G | 0.038 | 0.165 | 0.006 | 0.041 | 0.05 |
| NU-TE | BF | S2 | M | 0.079 | 0.079 | 0.008 | 0.045 | 0.057 |
| TE-NU | BF | S2 | M | 0.018 | 0.042 | 0.007 | 0.044 | 0.056 |
| NU-TE | BF | S2 | M+G | 0.138 | 0.191 | 0.006 | 0.045 | 0.058 |
| TE-NU | BF | S2 | M+G | 0.061 | 0.144 | 0.007 | 0.045 | 0.055 |
| NU-TE | BF | S3 | G | 0.110 | 0.192 | 0.007 | 0.033 | 0.04 |
| TE-NU | BF | S3 | G | 0.038 | 0.165 | 0.006 | 0.039 | 0.05 |
| NU-TE | BF | S3 | M | 0.304 | 0.299 | 0.007 | 0.031 | 0.036 |
| TE-NU | BF | S3 | M | 0.218 | 0.230 | 0.007 | 0.047 | 0.057 |
| NU-TE | BF | S3 | M+G | 0.334 | 0.370 | 0.008 | 0.049 | 0.059 |
| TE-NU | BF | S3 | M+G | 0.212 | 0.292 | 0.007 | 0.031 | 0.036 |
| NU-TE | IMF | S1 | G | 0.105 | 0.165 | 0.007 | 0.032 | 0.038 |
| TE-NU | IMF | S1 | G | 0.082 | 0.136 | 0.006 | 0.041 | 0.049 |
| NU-TE | IMF | S1 | M | -0.036 | -0.022 | 0.007 | 0.031 | 0.039 |
| TE-NU | IMF | S1 | M | -0.024 | -0.028 | 0.007 | 0.046 | 0.058 |
| NU-TE | IMF | S1 | M+G | 0.025 | 0.086 | 0.007 | 0.026 | 0.031 |
| TE-NU | IMF | S1 | M+G | 0.063 | 0.102 | 0.007 | 0.037 | 0.045 |
| NU-TE | IMF | S2 | G | 0.087 | 0.165 | 0.006 | 0.034 | 0.041 |
| TE-NU | IMF | S2 | G | 0.073 | 0.136 | 0.008 | 0.039 | 0.05 |
| NU-TE | IMF | S2 | M | 0.043 | 0.051 | 0.004 | 0.038 | 0.048 |
| TE-NU | IMF | S2 | M | -0.009 | 0.019 | 0.007 | 0.033 | 0.042 |
| NU-TE | IMF | S2 | M+G | 0.099 | 0.149 | 0.006 | 0.033 | 0.042 |
| TE-NU | IMF | S2 | M+G | 0.079 | 0.125 | 0.006 | 0.041 | 0.05 |
| NU-TE | IMF | S3 | G | 0.105 | 0.165 | 0.005 | 0.033 | 0.041 |
| TE-NU | IMF | S3 | G | 0.082 | 0.136 | 0.006 | 0.041 | 0.05 |
| NU-TE | IMF | S3 | M | 0.079 | 0.035 | 0.006 | 0.03 | 0.036 |
| TE-NU | IMF | S3 | M | -0.016 | -0.005 | 0.006 | 0.046 | 0.058 |
| NU-TE | IMF | S3 | M+G | 0.101 | 0.101 | 0.006 | 0.026 | 0.03 |
| TE-NU | IMF | S3 | M+G | 0.083 | 0.129 | 0.005 | 0.043 | 0.053 |
| NU-TE | LD | S1 | G | 0.068 | 0.097 | 0.007 | 0.026 | 0.031 |
| TE-NU | LD | S1 | G | 0.131 | 0.129 | 0.006 | 0.027 | 0.033 |
| NU-TE | LD | S1 | M | 0.008 | 0.002 | 0.008 | 0.041 | 0.053 |
| TE-NU | LD | S1 | M | 0.040 | 0.013 | 0.009 | 0.049 | 0.061 |
| NU-TE | LD | S1 | M+G | 0.040 | 0.068 | 0.007 | 0.041 | 0.051 |
| TE-NU | LD | S1 | M+G | 0.107 | 0.093 | 0.007 | 0.033 | 0.042 |
| NU-TE | LD | S2 | G | 0.068 | 0.097 | 0.006 | 0.025 | 0.029 |
| TE-NU | LD | S2 | G | 0.131 | 0.129 | 0.004 | 0.051 | 0.062 |
| NU-TE | LD | S2 | M | 0.027 | 0.032 | 0.006 | 0.029 | 0.036 |
| TE-NU | LD | S2 | M | 0.097 | 0.069 | 0.005 | 0.047 | 0.058 |
| NU-TE | LD | S2 | M+G | 0.064 | 0.078 | 0.005 | 0.044 | 0.052 |
| TE-NU | LD | S2 | M+G | 0.175 | 0.153 | 0.005 | 0.027 | 0.032 |
| NU-TE | LD | S3 | G | 0.068 | 0.097 | 0.005 | 0.029 | 0.034 |
| TE-NU | LD | S3 | G | 0.131 | 0.129 | 0.006 | 0.049 | 0.064 |
| NU-TE | LD | S3 | M | 0.034 | 0.014 | 0.006 | 0.03 | 0.036 |
| TE-NU | LD | S3 | M | 0.060 | 0.081 | 0.009 | 0.051 | 0.063 |
| NU-TE | LD | S3 | M+G | 0.052 | 0.057 | 0.007 | 0.044 | 0.054 |
| TE-NU | LD | S3 | M+G | 0.112 | 0.157 | 0.005 | 0.042 | 0.053 |
| NU-TE | LEA | S1 | G | 0.085 | 0.073 | 0.007 | 0.05 | 0.064 |
| TE-NU | LEA | S1 | G | 0.108 | 0.063 | 0.006 | 0.032 | 0.039 |
| NU-TE | LEA | S1 | M | 0.003 | -0.002 | 0.005 | 0.047 | 0.059 |
| TE-NU | LEA | S1 | M | 0.017 | -0.014 | 0.006 | 0.038 | 0.046 |
| NU-TE | LEA | S1 | M+G | 0.052 | 0.038 | 0.007 | 0.027 | 0.033 |
| TE-NU | LEA | S1 | M+G | 0.100 | 0.047 | 0.006 | 0.028 | 0.034 |
| NU-TE | LEA | S2 | G | 0.085 | 0.073 | 0.006 | 0.031 | 0.038 |
| TE-NU | LEA | S2 | G | 0.108 | 0.063 | 0.006 | 0.025 | 0.029 |
| NU-TE | LEA | S2 | M | -0.031 | -0.011 | 0.007 | 0.031 | 0.038 |
| TE-NU | LEA | S2 | M | 0.001 | 0.047 | 0.007 | 0.037 | 0.045 |
| NU-TE | LEA | S2 | M+G | 0.010 | 0.028 | 0.007 | 0.051 | 0.062 |
| TE-NU | LEA | S2 | M+G | 0.114 | 0.076 | 0.006 | 0.025 | 0.029 |
| NU-TE | LEA | S3 | G | 0.085 | 0.073 | 0.007 | 0.032 | 0.039 |
| TE-NU | LEA | S3 | G | 0.108 | 0.063 | 0.006 | 0.033 | 0.04 |
| NU-TE | LEA | S3 | M | 0.075 | 0.045 | 0.007 | 0.048 | 0.06 |
| TE-NU | LEA | S3 | M | 0.049 | 0.062 | 0.005 | 0.037 | 0.045 |
| NU-TE | LEA | S3 | M+G | 0.086 | 0.062 | 0.006 | 0.024 | 0.028 |
| TE-NU | LEA | S3 | M+G | 0.141 | 0.095 | 0.006 | 0.025 | 0.03 |

**Supplementary Table 2. Accuracy of prediction in permutation analysis.**

NU-TE= Training on Nucleus and predicting on Terminal. TE-NU= Training on Terminal and predicting on Nucleus.

S1= microbial collection at weaning. S2= microbial collection at mid-test. S3=microbial collection at off-test

M=Microbiome. G=Genome. M+G= Microbiome + Genome

BF= Back Fat. ADG= Average Daily Gain. LEA= Loin Area. LD= Loin Depth. IMF= Intramuscular fat.

Acc-Sout= Accuracy of prediction for Phenotype (Phe) for the Sire out (Sout) cross-validation

Acc-SSt= Accuracy of prediction for Phenotype (Phe) for the Sire stratified (SSt) cross-validation

Acc-Snull= Accuracy of prediction for Phenotype (Phe) for the permutation (Snull) cross-validation

Acc-SNull90%= 90^th^ percentile of Accuracy of prediction for Phenotype (Phe) for the permutation (Snull) cross-validation

Acc-SNull95%= 95^th^ percentile of Accuracy of prediction for Phenotype (Phe) for the permutation (Snull) cross-validation
